# Supplementary material for: Mapping the evolution of fertility support policies in China: A content and instrumental analysis
Source: PLoS One. 2025 Oct 9;20(10):e0332137. doi: 10.1371/journal.pone.0332137 (PMC12510515; doi:10.1371/journal.pone.0332137)
Supplement: S1 Appendix — (ZIP) [file pone.0332137.s001.zip › S1 Appendix. 226 original policy documents/126-卫生部、财政部关于印发《关于进一步加强农村孕产妇住院分娩工作的指导意见》的通知(FBM-CLI-4-112677).doc]

卫生部、财政部关于印发《关于进一步加强农村孕产妇住院分娩工作的指导意见》的通知

**发布部门：** [卫生部(已撤销)](https://www.pkulaw.com/law/chl?Aggs.IssueDepartment=60222) [财政部](https://www.pkulaw.com/law/chl?Aggs.IssueDepartment=60210) [机构沿革](javascript:void(0))

**发文字号：**卫妇社发〔2009〕12号

**发布日期：**2009.01.20

**实施日期：**2009.01.20

**时效性：** [现行有效](https://www.pkulaw.com/law/chl?Aggs.TimelinessDic=01)

**效力级别：** [部门规范性文件](https://www.pkulaw.com/law/chl?Aggs.EffectivenessDic=XE0302)

**法规类别：** [医疗保健](https://www.pkulaw.com/law/chl?Aggs.Category=09623) [妇幼卫生](https://www.pkulaw.com/law/chl?Aggs.Category=09622)

卫生部、财政部关于印发《关于进一步加强农村孕产妇住院分娩工作的指导意见》的通知
（卫妇社发[2009]12号）

各省、自治区、直辖市卫生厅局、财政厅局：
　　为落实温家宝总理在十一届全国人大一次会议《政府工作报告》中提出的在中西部地区农村实施住院分娩补助的政策，进一步提高农村孕产妇住院分娩率，降低孕产妇死亡率和婴儿死亡率，卫生部、财政部决定在全国加强农村孕产妇住院分娩工作。
　　现将《关于进一步加强农村孕产妇住院分娩工作的指导意见》印发你们，请认真贯彻落实。

卫生部
财政部
二OO九年一月二十日

关于进一步加强农村孕产妇住院分娩工作的指导意见

　　孕产妇死亡率和婴儿死亡率（以下简称两个死亡率）是一个国家经济和社会发展的重要指标，是联合国千年发展目标的重要内容。近年来，我国妇幼卫生事业有了长足的发展，孕产期保健服务水平不断提高，广大妇女儿童健康状况明显改善。但由于经济社会等多方面原因，我国农村特别是中西部农村地区住院分娩问题还没有得到很好地解决，两个死亡率仍处于较高水平。提高农村孕产妇住院分娩率是改善农村孕产妇保健状况、保障广大农村孕产妇和新生儿的生命安全、进一步降低两个死亡率的有效措施，对于提高农村人口的健康水平、促进社会的和谐与进步具有重要的现实意义和深远的历史意义。现就进一步加强农村孕产妇住院分娩工作提出以下指导意见。
　　**一、**指导思想、基本原则和工作目标
　　（一）指导思想。以邓小平理论和“三个代表”重要思想为指导，深入贯彻落实科学发展观，通过推进妇幼卫生服务网络建设、实施农村孕产妇住院分娩补助政策，使广大农村孕产妇能够平等享有安全、有效、规范、便捷的孕产期保健服务，维护广大农村妇女儿童的健康权益。
　　（二）基本原则。坚持以人为本，注重服务的公平性和可及性。坚持助产技术服务的区域规划，规范助产技术服务，保障孕产期保健服务质量。坚持政府主导，加大投入。
　　（三）工作目标。到2015年，东、中、西部地区各省（区、市）农村孕产妇住院分娩率分别达到95%、85%和80%以上；农村高危孕产妇住院分娩率达到95%以上；实现我国政府承诺的“联合国千年发展目标”。到2020年，东、中、西部各省（区、市）农村孕产妇住院分娩率分别达到98%、95%和90%以上；农村高危孕产妇住院分娩率达到98%以上；孕产妇死亡率和婴儿死亡率达到中等发达国家水平。

　　**二、**主要内容
　　（四）明确农村孕产妇住院分娩服务项目和标准。卫生部会同财政部制订农村孕产妇住院分娩的基本服务项目。在此基础上，各省（区、市）结合本地经济社会发展水平，制订本地区的服务项目和限价标准，保证住院分娩服务质量与安全。
　　（五）完善农村孕产妇住院分娩服务体系。以县级妇幼保健机构为区域内孕产期保健技术指导和管理中心，提高县乡两级孕产期保健服务能力。建立健全以各级妇幼保健机构为主体，县乡两级医疗卫生机构为基础，其他医疗卫生机构为支撑，基本覆盖全国城乡的妇幼保健服务网络，为孕产妇享有规范、安全的孕产期保健及住院分娩服务提供保障。
　　（六）加强医疗卫生机构和人员服务资质的监督管理。加强助产技术服务的区域规划，严格助产机构、技术和人员准入，引导孕产妇到有资质的医疗卫生机构（包括社会力量举办的医疗卫生机构）住院分娩，坚决打击各种非法接生行为。
　　（七）强化产科质量管理。健全医疗卫生机构产科质量管理体系，按照孕产期保健和助产技术规范，制定质量控制方案和质量评估标准，定期开展质量评估活动，不断改进产科服务质量。各地要规范高危孕产妇筛查、转诊和救治流程，建立危重孕产妇抢救绿色通道；严格剖宫产指征，控制剖宫产率，降低医疗风险；加大产科质量督查力度，实行周期性与日常督查相结合，督促助产机构规范医疗行为，努力保障产科质量，维护广大孕产妇生命安全。
　　（八）实施农村孕产妇住院分娩补助政策。在各地核定成本、明确限价标准的基础上，对农村孕产妇住院分娩所需费用予以财政补助，补助标准由各省（区、市）财政部门会同卫生部门制定。参加新型农村合作医疗的农村孕产妇在财政补助之外的住院分娩费用，可按当地新型农村合作医疗制度的规定给予补偿。对个人负担较重的贫困孕产妇，可由农村医疗救助制度按规定给予救助。鼓励有条件的地区探索将农村孕产妇住院分娩补助与新型农村合作医疗、农村医疗救助补助统筹管理使用。

　　**三、**保障措施
　　（九）卫生部负责研究制定技术规范、考核标准，财政部负责制定资金管理办法等配套政策。各地卫生、财政等相关部门负责制定具体实施办法并组织实施。
　　（十）中央财政对困难地区农村孕产妇住院分娩给予补助。地方财政也应承担相应的支出责任，并统筹使用各级财政补助资金，按照国家专项补助资金管理的规定加强管理，专款专用，保障资金安全，发挥专项资金效益。
　　（十一）加强人员培训，改善住院分娩基础设备，提高农村基层医疗卫生机构服务能力。本着经常性、基础性、实用性的原则，采取多种方式开展基本知识、基本理论、基本技能等方面的培训，提高县乡两级助产人员的服务能力。
　　（十二）县级卫生部门要会同有关部门加强农村孕产妇住院分娩工作的监督管理。健全农村孕产妇住院分娩补助工作规程，做好农村孕产妇基础信息统计和管理工作。建立并完善考核评价制度，严格管理和监督。

　　**四、**组织领导
　　（十三）各级政府要以高度的政治责任感和使命感加强组织领导，认真贯彻落实住院分娩的各项政策。根据实际情况，制定实施方案，明确责任，统筹协调，督查指导，推动农村住院分娩工作持续健康发展。
　　（十四）做好农村孕产妇住院分娩补助政策宣传和孕产期保健的健康教育工作，做到家喻户晓，为孕产妇住院分娩营造良好的社会氛围。
　　附件：

住院分娩基本服务项目

　　**一、**正常产
　　（一）基本护理
　　床位，护理（一般护理、产前护理、产后护理）。
　　（二）常规检查
　　1．一般检查：体格检查、产科检查。
　　2．辅助检查：血常规、凝血功能（凝血时间）、肝功能、乙肝两对半、尿常规等。
　　（三）接产服务
　　1．观察产程：测血压、听胎心、查胎位、宫缩、阴道检查。
　　2．接生。
　　3．新生儿护理：脐带处理、呼吸道清理、新生儿体检等。
　　4．新生儿窒息复苏（如发生）。
　　5．产房观察：血压、脉搏；宫缩、宫底高度、阴道出血；新生儿一般情况。
　　（四）基本药物
　　缩宫素、生理盐水（或葡萄糖注射液）、抗生素、眼药。

　　**二、**阴道手术助产
　　（一）基本护理
　　床位，护理（一般护理、产前护理、产后护理）。
　　（二）常规检查
　　1．一般检查：体格检查、产科检查。
　　2．辅助检查：血常规、凝血功能（凝血时间）、肝功能、乙肝两对半、尿常规等。
　　（三）接产服务
　　1．观察产程：测血压、听胎心、查胎位、宫缩、阴道检查。
　　2．阴道助产：会阴切开与缝合术、胎头吸引术、产钳术、臀牵引术、臀助产术。
　　3．新生儿护理：脐带处理、呼吸道清理、新生儿体检等。
　　4．新生儿窒息复苏（如发生）。
　　5．产房观察：血压、脉搏；宫缩、宫底高度、阴道出血；新生儿一般情况。
　　（四）基本药物
　　缩宫素、生理盐水（或葡萄糖注射液）、抗生素、眼药。

　　**三、**剖宫产
　　（一）基本护理
　　床位，护理（一般护理、术前护理、术后护理）。
　　（二）常规检查
　　1．一般检查：体格检查、产科检查。
　　2．辅助检查：血常规、凝血功能（凝血时间）、交叉配血、肝功能、乙肝两对半、尿常规、心电图检查等。
　　（三）接产服务
　　1．观察产程：听胎心、测血压、查胎位、宫缩、阴道检查。
　　2．剖宫产手术。
　　3．新生儿护理：脐带处理、呼吸道清理、新生儿体检等。
　　4．新生儿窒息复苏（如发生）。
　　（四）基本药物
　　缩宫素、止血芳酸（或止血敏）、抗生素、生理盐水（或葡萄糖注射液）、眼药。

© 北大法宝：（[www.pkulaw.com](https://www.pkulaw.com/)) 专业提供法律信息、法学知识和法律软件领域各类解决方案。北大法宝为您提供丰富的参考资料，正式引用法规条文时请与标准文本核对。
欢迎查看所有[产品和服务](https://www.pkulaw.com/helps/2.html)。法宝快讯：[如何快速找到您需要的检索结果？](https://www.pkulaw.com/helps/70.html)[法宝](https://www.pkulaw.com/helps/69.html)V6有何新特色？

扫描二维码阅读原文

**原文链接：**<https://www.pkulaw.com/chl/f4a349efdd46402cbdfb.html>
